# Addendum to: Emerging patterns of New Physics with and without Lepton Flavour Universal contributions

**DOI:** 10.1140/epjc/s10052-020-8018-3

**Published:** 2020-06-08

**Authors:** Marcel Algueró, Bernat Capdevila, Andreas Crivellin, Sébastien Descotes-Genon, Pere Masjuan, Joaquim Matias, Martín Novoa-Brunet, Javier Virto

**Affiliations:** 1 Grup de Física Teòrica, Departament de Física, Universitat Autònoma de Barcelona, 08193 Bellaterra, Barcelona Spain; 2 Institut de Física d’Altes Energies (IFAE), The Barcelona Institute of Science and Technology, Campus UAB, 08193 Bellaterra, Barcelona Spain; 3 Università di Torino and INFN Sezione di Torino, Via P. Giuria 1, Torino, I-10125 Italy; 4 Paul Scherrer Institut, 5232 Villigen PSI, Switzerland; 5 Physik-Institut, Universität Zürich, Winterthurerstrasse 190, 8057 Zürich, Switzerland; 6 Laboratoire de Physique Théorique, UMR 8627, CNRS, Univ. Paris-Sud, Université Paris-Saclay, 91405 Orsay Cedex, France; 7 Université Paris-Saclay, CNRS/IN2P3, IJCLab, 91405 Orsay, France; 8 Physics Department T31, Technische Universität München, James Franck-Strasse 1, 85748 Garching, Germany; 9 Center for Theoretical Physics, Massachusetts Institute of Technology, 77 Massachusetts Av., Cambridge, MA 02139 USA

## Abstract

This paper is an addendum to Ref. Eur. Phys. J. C79 (2019) no. 8, 714 that supersedes all results (Tables and Figures) in that paper after including the new data on the $B\rightarrow K^*\mu \mu$ angular distribution released in 2020 by the LHCb collaboration. The new results confirm all the conclusions in Eur. Phys. J. C79 (2019) no. 8, 714, exhibiting an increase in the coherence and significance of the hypotheses and confirming the existence of a puzzle.

## Addendum to: Eur. Phys. J. C10.1140/epjc/s10052-019-7216-3

## State-of-the-art $b \rightarrow s \ell \ell$ global fits in March 2020

This addendum updates the results presented in Ref. [1] and in Ref. [2] after including the most recent $B \rightarrow K^*\mu \mu$ angular distribution data from the LHCb collaboration [3], released in March 2020. As such, the tables and figures presented in the following supersede the ones in Ref. [1]:

- Figures 1,  2,  3,  4,  5 (left), 5 (right) in Ref. [1] are superseded by Figs. 1, 5 (left), 3, 4, 5 (right), 2 (left), respectively.
- Tables I, II, III, and V in Ref. [1] are superseded by Tables 2, 3, 4, and 5, respectively.
- Figures 2 (right) and 6 are new.

**Table 1:** List of updated input parameters in the present analysis

Re $\lambda _u$	$3.383 \times 10^{-4}$
Im $\lambda _u$	$7.555 \times 10^{-4}$
$\lambda _t$	$(4.124 \pm 0.063)\times 10^{-2}$
$\tau _{B^0}$	$1.520 \times 10^{-12}$ s
$\tau _{B^+}$	$1.638 \times 10^{-12}$ s
$\tau _{B_s}$	$1.509 \times 10^{-12}$ s

**Table 2:** Most prominent 1D patterns of NP in $b\rightarrow s\mu ^+\mu ^-$ transitions (state-of-the-art fits as of March 2020). Here, Pull$_\mathrm{SM}$ is quoted in units of standard deviation and the *p*-value of the SM hypothesis is 1.4% for the fit “All” and 12.6% for the fit LFUV

	All				LFUV			
1D Hyp.	Best fit	1 $\sigma$/2 $\sigma$	Pull$_\mathrm{SM}$	p-value	Best fit	1 $\sigma$/ 2 $\sigma$	Pull$_\mathrm{SM}$	p-value
${{\mathcal {C}}}_{9\mu }^\mathrm{NP}$	$-$1.03	$[-1.19,-0.88]$	6.3	37.5 %	$-$0.91	$[-1.25,-0.61]$	3.3	60.7 %
		$[-1.33,-0.72]$				$[-1.63,-0.34]$		
${{\mathcal {C}}}_{9\mu }^\mathrm{NP}=-{{\mathcal {C}}}_{10\mu }^\mathrm{NP}$	$-$0.50	$[-0.59,-0.41]$	5.8	25.3 %	$-$0.39	$[-0.50,-0.28]$	3.7	75.3 %
		$[-0.69,-0.32]$				$[-0.62,-0.17]$		
${{\mathcal {C}}}_{9\mu }^\mathrm{NP}=-{{\mathcal {C}}}_{9'\mu }$	$-$1.02	$[-1.17,-0.87]$	6.2	34.0 %	$-$1.67	$[-2.15,-1.05]$	3.1	53.1 %
		$[-1.31,-0.70]$				$[-2.54,-0.48]$		
${{\mathcal {C}}}_{9\mu }^\mathrm{NP}=-3 {{\mathcal {C}}}_{9e}^\mathrm{NP}$	$-$0.93	$[-1.08,-0.78]$	6.2	33.6 %	$-$0.68	$[-0.92,-0.46]$	3.3	60.8 %
		$[-1.23,-0.63]$				$[-1.19,-0.25]$		

The data presented in Ref. [3] corresponds to an integrated luminosity of 4.7 $\hbox {fb}^{-1}$ collected by LHCb collaboration. Our global analysis now includes 180 observables corresponding to: (i) all previous data [4] (ii) updates discussed in Ref. [1] and (iii) the combined Run1+2016 data for optimized observables presented in Ref. [3]. The combined Run1+2016 data share two main features: on the one hand, the global picture is very coherent with respect to the Run-1 and part of Run-2 (2015-2016) data used in Ref. [1]. On the other hand, errors are generally reduced, specially in the bins [1.1, 2.5] and [2.5, 4.0]. These two features, by themselves, will reduce the p-value of the SM as we will see below.

In the analysis presented in this addendum, besides updating the data, we have also updated some input parameters (see Table 1) and improved the theoretical prediction for $B_s \rightarrow \mu \mu$ using the results from Refs. [5, 6]. However, it turns out that this theory update has a relatively marginal impact on our results.

**Table 3:** Most prominent 2D patterns of NP in $b\rightarrow s\mu ^+\mu ^-$ transitions (state-of-the-art fits as of March 2020). The last five rows correspond to Hypothesis 1: $({{\mathcal {C}}}_{9\mu }^\mathrm{NP}=-{{\mathcal {C}}}_{9^\prime \mu } , {{\mathcal {C}}}_{10\mu }^\mathrm{NP}={{\mathcal {C}}}_{10^\prime \mu })$, 2: $({{\mathcal {C}}}_{9\mu }^\mathrm{NP}=-{{\mathcal {C}}}_{9^\prime \mu } , {{\mathcal {C}}}_{10\mu }^\mathrm{NP}=-{{\mathcal {C}}}_{10^\prime \mu })$, 3: $({{\mathcal {C}}}_{9\mu }^\mathrm{NP}=-{{\mathcal {C}}}_{10\mu }^\mathrm{NP} , {{\mathcal {C}}}_{9^\prime \mu }={{\mathcal {C}}}_{10^\prime \mu }$), 4: $({{\mathcal {C}}}_{9\mu }^\mathrm{NP}=-{{\mathcal {C}}}_{10\mu }^\mathrm{NP} , {{\mathcal {C}}}_{9^\prime \mu }=-{{\mathcal {C}}}_{10^\prime \mu })$ and 5: $({{\mathcal {C}}}_{9\mu }^\mathrm{NP} , {{\mathcal {C}}}_{9^\prime \mu }=-{{\mathcal {C}}}_{10^\prime \mu })$

	All			LFUV		
2D Hyp.	Best fit	$\hbox {Pull}_\mathrm{SM}$	p-value	Best fit	$\hbox {Pull}_\mathrm{SM}$	p-value
$({{\mathcal {C}}}_{9\mu }^\mathrm{NP},\;{{\mathcal {C}}}_{10\mu }^\mathrm{NP})$	($-$0.98,+0.19)	6.2	39.8%	($-$0.31,+0.44)	3.2	70.0%
$({{\mathcal {C}}}_{9\mu }^\mathrm{NP},\;{{\mathcal {C}}}_{7^{\prime }})$	($-$1.04,+0.01)	6.0	36.5%	($-$0.92,$-$0.04)	3.0	57.4%
$({{\mathcal {C}}}_{9\mu }^\mathrm{NP},\;{{\mathcal {C}}}_{9^\prime \mu })$	($-$1.14,+0.55)	6.5	47.4%	($-$1.86,+1.20)	3.5	81.2%
$({{\mathcal {C}}}_{9\mu }^\mathrm{NP},\;{{\mathcal {C}}}_{10^\prime \mu })$	($-$1.17,$-$0.33)	6.6	50.3%	($-$1.87,$-$0.59)	3.7	89.6%
$({{\mathcal {C}}}_{9\mu }^\mathrm{NP},\; {{\mathcal {C}}}_{9e}^\mathrm{NP})$	($-$1.09,$-$0.25)	6.0	36.5%	($-$0.72,+0.19)	2.9	54.5%
Hyp. 1	($-$1.10,+0.28)	6.5	48.9%	($-$1.69,+0.29)	3.5	82.4%
Hyp. 2	($-$1.01,+0.07)	5.9	33.7%	($-$1.95,+0.22)	3.1	64.3%
Hyp. 3	($-$0.51,+0.10)	5.4	24.0%	($-$0.39,$-$0.04)	3.2	69.9%
Hyp. 4	($-$0.52,+0.11)	5.6	26.4%	($-$0.46,+0.15)	3.4	77.9%
Hyp. 5	($-$1.17,+0.23)	6.6	51.1%	($-$2.05,+0.50)	3.8	91.9%

**Table 4:** 1 and $2\sigma$ confidence intervals for the NP contributions to Wilson coefficients in the 6D hypothesis allowing for NP in $b\rightarrow s\mu ^+\mu ^-$ operators dominant in the SM and their chirally-flipped counterparts, for the fit “All” (state-of-the-art as of March 2020). The $\hbox {Pull}_\mathrm{SM}$ is $5.8\sigma$ and the *p*-value is $46.8 \%$

	${{\mathcal {C}}}_{7}^\mathrm{NP}$	${{\mathcal {C}}}_{9\mu }^\mathrm{NP}$	${{\mathcal {C}}}_{10\mu }^\mathrm{NP}$	${{\mathcal {C}}}_{7^\prime }$	${{\mathcal {C}}}_{9^\prime \mu }$	${{\mathcal {C}}}_{10^\prime \mu }$
Best fit	+0.00	$-$1.13	+0.20	+0.00	+0.49	$-$0.10
1 $\sigma$	$[-0.02,+0.02]$	$[-1.30,-0.96]$	$[+0.05,+0.37]$	$[-0.01,+0.02]$	$[+0.04,+0.95]$	$[-0.33,+0.14]$
2 $\sigma$	$[-0.03,+0.04]$	$[-1.46,-0.78]$	$[-0.09,+0.57]$	$[-0.03,+0.04]$	$[-0.39,+1.45]$	$[-0.55,+0.41]$

**Fig. 1:**
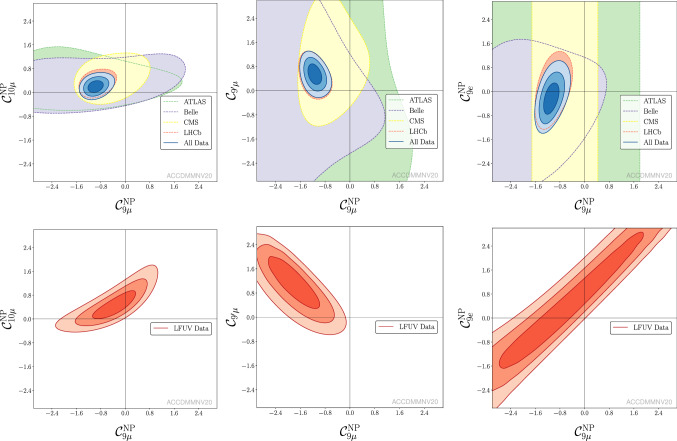
From left to right: allowed regions in the $({{\mathcal {C}}}_{9\mu }^\mathrm{NP},{{\mathcal {C}}}_{10\mu }^\mathrm{NP})$, $({{\mathcal {C}}}_{9\mu }^\mathrm{NP},{{\mathcal {C}}}_{9^\prime \mu })$ and $({{\mathcal {C}}}_{9\mu }^\mathrm{NP},{{\mathcal {C}}}_{9e}^\mathrm{NP})$ planes for the corresponding 2D hypotheses, using all available data (fit “All”) upper row or LFUV fit lower row

**Table 5:** Most prominent patterns for LFU and LFUV NP contributions from Fit “All” (state-of-the-art as of March 2020). See Table V of Ref. [1] for more detail

Scenario		Best-fit point	1 $\sigma$	2 $\sigma$	$\hbox {Pull}_\mathrm{SM}$	p-value
Scenario 5	${{\mathcal {C}}}_{9\mu }^\mathrm{V}$	$-0.54$	$[-1.06,-0.06]$	$[-1.68,+0.39]$	6.0	39.4 %
	${{\mathcal {C}}}_{10\mu }^\mathrm{V}$	$+0.58$	$[+0.13,+0.97]$	$[-0.48,+1.33]$		
	${{\mathcal {C}}}_{9}^\mathrm{U}={{\mathcal {C}}}_{10}^\mathrm{U}$	$-0.43$	$[-0.85,+0.05]$	$[-1.23,+0.67]$		
Scenario 6	${{\mathcal {C}}}_{9\mu }^\mathrm{V}=-{{\mathcal {C}}}_{10\mu }^\mathrm{V}$	$-0.56$	$[-0.65,-0.47]$	$[-0.75,-0.38]$	6.2	41.4 %
	${{\mathcal {C}}}_{9}^\mathrm{U}={{\mathcal {C}}}_{10}^\mathrm{U}$	$-0.41$	$[-0.53,-0.29]$	$[-0.64,-0.16]$		
Scenario 7	${{\mathcal {C}}}_{9\mu }^\mathrm{V}$	$-0.84$	$[-1.15,-0.54]$	$[-1.48,-0.26]$	6.0	36.5 %
	${{\mathcal {C}}}_{9}^\mathrm{U}$	$-0.25$	$[-0.59,+0.10]$	$[-0.92,+0.47]$		
Scenario 8	${{\mathcal {C}}}_{9\mu }^\mathrm{V}=-{{\mathcal {C}}}_{10\mu }^\mathrm{V}$	$-0.34$	$[-0.44,-0.25]$	$[-0.54,-0.16]$	6.5	48.4 %
	${{\mathcal {C}}}_{9}^\mathrm{U}$	$-0.80$	$[-0.98,-0.60]$	$[-1.16,-0.39]$		
Scenario 9	${{\mathcal {C}}}_{9\mu }^\mathrm{V}=-{{\mathcal {C}}}_{10\mu }^\mathrm{V}$	$-0.66$	$[-0.79,-0.52]$	$[-0.93,-0.40]$	5.7	28.4 %
	${{\mathcal {C}}}_{10}^\mathrm{U}$	$-0.40$	$[-0.63,-0.17]$	$[-0.86,+0.07]$		
Scenario 10	${{\mathcal {C}}}_{9\mu }^\mathrm{V}$	$-1.03$	$[-1.18,-0.87]$	$[-1.33,-0.71]$	6.2	41.5 %
	${{\mathcal {C}}}_{10}^\mathrm{U}$	$+0.28$	$[+0.12,+0.45]$	$[-0.04,+0.62]$		
Scenario 11	${{\mathcal {C}}}_{9\mu }^\mathrm{V}$	$-1.11$	$[-1.26,-0.95]$	$[-1.40,-0.78]$	6.3	43.9 %
	${{\mathcal {C}}}_{10'}^\mathrm{U}$	$-0.29$	$[-0.44,-0.15]$	$[-0.58,-0.01]$		
Scenario 12	${{\mathcal {C}}}_{9'\mu }^\mathrm{V}$	$-0.06$	$[-0.21,+0.10]$	$[-0.37,+0.26]$	2.1	2.2 %
	${{\mathcal {C}}}_{10}^\mathrm{U}$	$+0.44$	$[+0.26,+0.62]$	$[+0.09,+0.81]$		
Scenario 13	${{\mathcal {C}}}_{9\mu }^\mathrm{V}$	$-1.16$	$[-1.31,-1.00]$	$[-1.46,-0.83]$	6.2	49.2 %
	${{\mathcal {C}}}_{9'\mu }^\mathrm{V}$	$+0.56$	$[+0.27,+0.83]$	$[-0.02,+1.10]$		
	${{\mathcal {C}}}_{10}^\mathrm{U}$	$+0.28$	$[+0.08,+0.49]$	$[-0.11,+0.70]$		
	${{\mathcal {C}}}_{10'}^\mathrm{U}$	$+0.01$	$[-0.19,+0.22]$	$[-0.40,+0.42]$		

### Theoretical update of $B_s \rightarrow \mu \mu$

Our analyses include ${{\mathcal {B}}}(B_s \rightarrow \mu ^+\mu ^-)$ as it constrains the space for NP contributions in ${{\mathcal {C}}}_{10\mu }$ and $\mathcal{C}_{10'\mu }$ significantly. The expression of this branching ratio can be derived from Ref. [7] taking into account that we use $\mathcal{O}_{10\ell }=\frac{e^2}{16\pi ^2}({\bar{s}}\gamma _{\mu }P_{L}b)(\bar{\ell }\gamma ^{\mu }\gamma _5\ell )$, where ${P_{L}=\frac{1 {-}\gamma _5}{2}}$, instead of the axial operator $\mathcal{O}_{A}=({\bar{b}}\gamma _{\mu }s)(\bar{\ell }\gamma ^{\mu }\gamma _5\ell )$ used in Ref. [7] and therefore reads

1 $$\begin{aligned} {{\mathcal {B}}}_{B_s\rightarrow \mu \mu }^\mathrm{SM}=\frac{\lambda ^2_t G^2_F m^3_{B_s} \alpha ^2_{em} \tau _{B_s} f^2_{B_s}}{64\pi ^3} r^2\sqrt{1-r^2}|\mathcal{C}_{10\mu }^\mathrm{SM}|^2\,, \end{aligned}$$

where $r={2m_{\mu }}/{m_{B_s}}$. Once NP contributions in ${{\mathcal {C}}}_{10}$ and in the chirality-flipped Wilson coefficient ${{\mathcal {C}}}_{10^\prime }$ are included, the full expression in our analyses, excluding scalar and pseudoscalar operators, reads

2 $$\begin{aligned} {{\mathcal {B}}}_{B_s\rightarrow \mu \mu }=\frac{\lambda ^2_t G^2_F m^3_{B_s} \alpha ^2_{em} \tau _{B_s} f^2_{B_s}}{64\pi ^3}r^2\sqrt{1-r^2} (\mathcal{C}_{10\mu }-{{\mathcal {C}}}_{10'\mu })^2 \end{aligned}$$

where $${{\mathcal {C}}}_{10\mu }={{\mathcal {C}}}^\mathrm{{SM}}_{10\mu }+\mathcal{C}^\mathrm{{NP}}_{10\mu }$$.

As discussed in Refs. [8, 9], the LHCb measurement of $B_s$ decays is performed after integrating the time evolution of the $B_s$ meson and its mixing with ${\bar{B}}_s$. The resulting correction is an effect of $O(\Delta \Gamma _s/\Gamma _s)$ and it is modulated by an asymmetry $A_{\Delta \Gamma }$ which depends on the process considered. In the SM, for $B_s\rightarrow \mu \mu$, this asymmetry is known to be +1 [9]: the time-integrated branching ratio $\overline{{{\mathcal {B}}}}_{s\mu \mu }$ is then obtained from $\mathcal{B}_{B_s\rightarrow \mu \mu }$ by replacing the average of the lifetimes of the light and heavy mass eigenstates $\tau _{B_s}$ by that of the heavy mass eigenstate $\tau ^s_{H}$ (see for instance the assessment performed in Ref. [7] within the SM). The asymmetry $A_{\Delta \Gamma }$ can be changed in the presence of NP contributions to ${{\mathcal {C}}}_{10'\mu }$, inducing an a priori different $O(\Delta \Gamma _s/\Gamma _s)$ correction from time integration.^1^ In principle we should thus enlarge the error on the prediction of $\bar{{\mathcal {B}}}_{B_s\rightarrow \mu \mu }$ in the case of scenarios involving NP in ${{\mathcal {C}}}_{10'\mu }$ to take into account the uncertainty on the $O(\Delta \Gamma _s/\Gamma _s)$ correction. We checked explicitly that enlarging this uncertainty has no actual impact on the outcome of the fits and for simplicity we will thus keep the SM uncertainty on $\bar{\mathcal{B}}_{B_s\rightarrow \mu \mu }$ for all our analyses.

The most recent theoretical prediction for ${{\mathcal {B}}}_{B_s\rightarrow \mu \mu }$ includes a set of electromagnetic corrections at scales below $m_b$ that are dynamically enhanced by $m_b/\Lambda _\mathrm{{QCD}}$ and by large logarithms [5]. The size of such corrections, found to be $1\%$, is larger than previous estimates of next-to-leading order QED effects, assessed to be $\pm 0.3\%$. To account for these new corrections, we have rescaled our theoretical prediction Eq. (2) by an overall factor $\Delta _{B_{s\mu \mu }}$ so that our own set of input parameters yields an SM result in agreement with the value presented at the Orsay workshop in 2019 [6]:

3 $$\begin{aligned} \overline{{{\mathcal {B}}}}_{s\mu \mu }^\mathrm{SM}=\eta _\mathrm{{QED}}(3.65\pm 0.23) \times 10^{-9}=(3.64\pm 0.14) \times 10^{-9}. \end{aligned}$$

where the effect of the QED corrections from Ref. [5] is introduced as a global factor $\eta _\mathrm{{QED}}=0.993$.

### Updated 1D, 2D and 6D global fits to $b\rightarrow s \ell \ell$ flavour anomalies in March 2020

Tables 2, 3 and 4 collect the updated results for the most prominent LFUV NP scenarios. These tables (updated using March 2020 data) supersede the ones presented in in Ref. [1], i.e. Tabs. I, II and III, respectively. A discussion on the most relevant NP scenarios can be found in Ref. [1]. Figure 1 provide a graphical account of the most remarkable results.

Table 5 collects the updated NP scenarios combining LFUV and LFU, thus superseding the results presented in Ref. [1] (Table V) and those presented in Ref. [2]. Among the scenarios presented in this table, we find one of the most significant solutions in terms of sigmas (scenario 8) as can also be seen in Figs. 3 and 4.

**Fig. 2:**
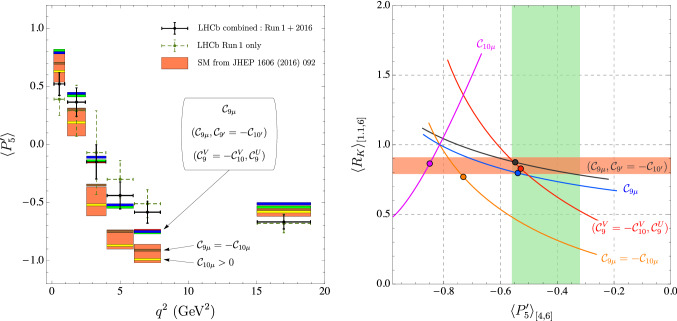
Left: impact of favoured NP scenarios on the observable $P_5^\prime$. This figure supersedes Fig. 5 in Ref. [1]. Only central values for the NP scenarios are displayed. The most interesting scenarios cluster together, ${{\mathcal {C}}}_{9\mu }^\mathrm{NP}$ in red, $({{\mathcal {C}}}_{9\mu }^\mathrm{NP},{{\mathcal {C}}}_{9'\mu }^\mathrm{NP}=-{{\mathcal {C}}}_{10'\mu }^\mathrm{NP})$ in green and $({{\mathcal {C}}}_{9}^\mathrm{V}=-{{\mathcal {C}}}_{10}^\mathrm{V},{{\mathcal {C}}}_9^\mathrm{U})$ in blue, and they are now in better agreement with $P'_5$ data. On the other hand, ${{\mathcal {C}}}_{9\mu }^\mathrm{NP}=-\mathcal{C}_{10\mu }^\mathrm{NP}$ (brown) and ${{\mathcal {C}}}_{10\mu }^\mathrm{NP}$ (yellow, with a global significance of only 3.2$\sigma$) fail to explain the deviations observed for this observable. Right: $\langle R_K \rangle _{[1.1,6]}$ versus $\langle P_5' \rangle _{[4,6]}$ in five different scenarios: ${{\mathcal {C}}}_{9\mu }^\mathrm{NP}$ (blue), $\mathcal{C}_{9\mu }^\mathrm{NP} = - {{\mathcal {C}}}_{10\mu }^\mathrm{NP}$ (orange), and $({{\mathcal {C}}}^\mathrm{V}_{9\mu }= - {{\mathcal {C}}}^\mathrm{V}_{10\mu }, {{\mathcal {C}}}^\mathrm{U}_{9})$ (red), $({{\mathcal {C}}}_{9\mu }^\mathrm{NP}, {{\mathcal {C}}}_{9'\mu }^\mathrm{NP}= - {{\mathcal {C}}}_{10'\mu }^\mathrm{NP})$ (black), and $\mathcal{C}_{10\mu }^\mathrm{NP}$ (pink). This figure partially supersedes Fig. 12 in Ref. [10]. The curves correspond only to the predictions for central values. In the 2D scenarios (red and black) the Wilson coefficient not shown is set to its b.f.p. value. The current experimental values from the LHCb collaboration are also indicated (orange horizontal and green vertical bands respectively). The dots correspond to the b.f.p. values of the corresponding scenario for the fit to the “All” data set

**Fig. 3:**
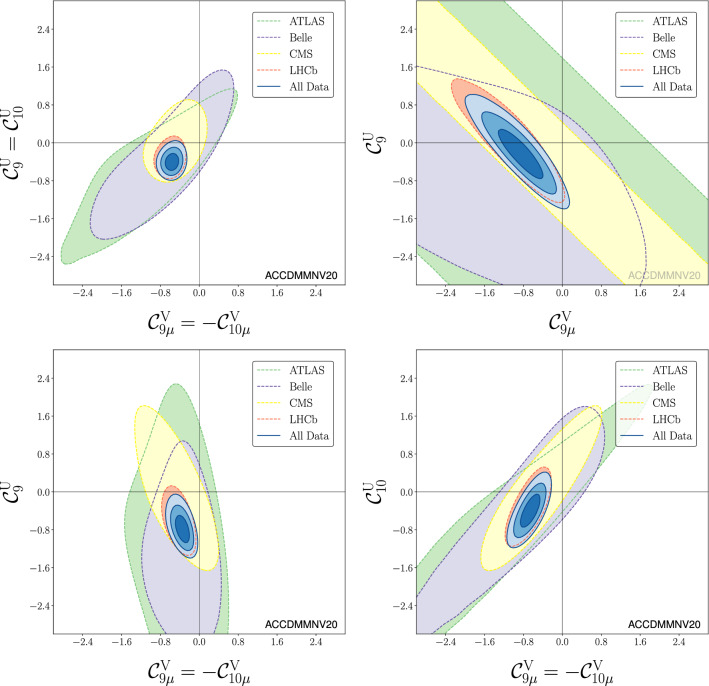
Updated plots of Ref. [2] corresponding to Scenarios 6, 7, 8, 9

**Fig. 4:**
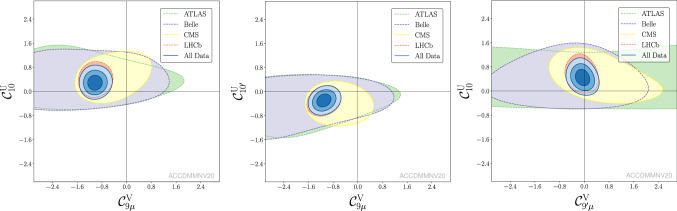
Updated plots of Ref. [2] corresponding to the Scenarios 10, 11, 12

**Fig. 5:**
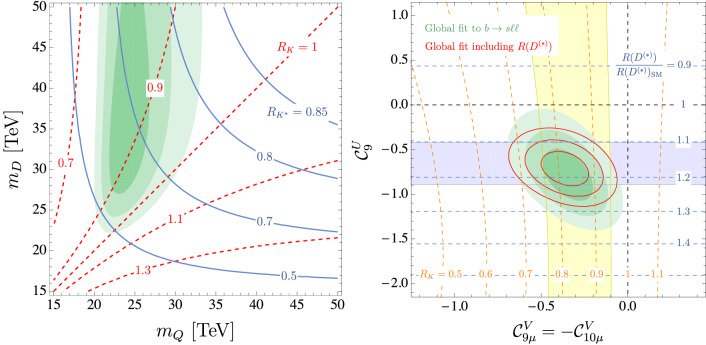
Left: This plot supersedes Fig. 2 from Ref. [1] and describes the preferred regions (at the 1, 2 and 3$\,\sigma$ level) for the $L_\mu -L_\tau$ model of Ref. [16] from $b\rightarrow s\ell ^+\ell ^-$ data (green) in the $(m_Q,\, m_D)$ plane with $Y^{D,Q}=1$. The contour lines denote the predicted values for $R_K^{[1.1,6]}$ (red, dashed) and $R_{K^*}^{[1.1,6]}$ (blue, solid). Right: This plot supersedes the left plot in Fig. 5 and it represents the preferred regions at the 1, 2 and 3$\,\sigma$ level (green) in the $({{\mathcal {C}}}_{9\mu }^\mathrm{V}=-{{\mathcal {C}}}_{10\mu }^\mathrm{V},\,{{\mathcal {C}}}_{9}^\mathrm{U})$ plane from $b\rightarrow s\ell ^+\ell ^-$ data. The red contour lines show the corresponding regions once $R_{D^{(*)}}$ is included in the fit (for $\Lambda =2$ TeV). The horizontal blue (vertical yellow) band is consistent with $R_{D^{(*)}}$ ($R_{K}$) at the $2\,\sigma$ level and the contour lines show the predicted values for these ratios

**Fig. 6:**
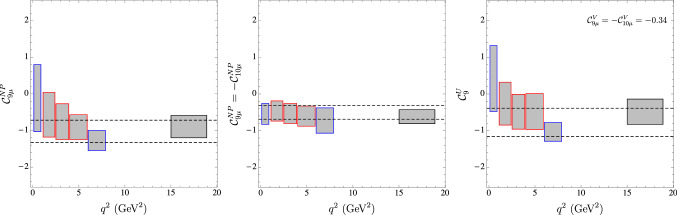
Determination of Wilson coefficients in a bin-by-bin fit using only the new LHCb data on optimized observables, branching ratios and radiative decays. Each box correspond to the 1$\sigma$ confidence interval obtained in this bin. Left: $\mathcal{C}_{9\mu }^\mathrm{NP}$ assuming NP affects only this Wilson coefficient. Middle: ${{\mathcal {C}}}_{9\mu }^\mathrm{NP}=-\mathcal{C}_{10\mu }^\mathrm{NP}$ assuming NP affects only these Wilson coefficients. Right: ${{\mathcal {C}}}_{9}^\mathrm{U}$ in scenario 8, setting the LFUV coefficients ${{\mathcal {C}}}_{9\mu }^\mathrm{V}=-\mathcal{C}_{10\mu }^\mathrm{V}$ to their values at the best-fit point of the “All” fit. In each case, the band corresponds to the 2$\sigma$ interval obtained from the fit of the NP hypothesis to the “All” data set

We observe an excellent consistency between the previous and the new data. This is a remarkable fact since almost 50 angular observables have been updated in the most recent LHCb collaboration analysis with uncertainty reductions of $30-50\%$ or more (in particular for the bins [1.1, 2.5] and [2.5, 4]). The consistency between all observables previously observed is confirmed with a slightly increased tension (bin by bin) compared to the SM in basically all angular observables. New tensions with respect to the SM appear in $\langle P_3 \rangle _{[1.1,2.5]}$, $\langle P'_6 \rangle _{[6,8]}$ and $\langle P'_8 \rangle _{[1.1,2.5]}$. The tension in the first bin of $P'_5$ has decreased and it is now more similar in size with respect to other tensions [10] (with the caveat that the experimental analysis relies on an expression of the angular distribution holding in the massless limit, which might bias the analysis in this first bin). The pull of $\langle P'_4 \rangle _{[4,6]}$ has changed sign so that $\langle P'_4 \rangle _{[4,6]}$ and $\langle P_1 \rangle _{[4,6]}$ are not anymore in tension, favouring a contribution to ${{\mathcal {C}}}_{10'\mu }$ (see Table 3).

Following this increased consistency, there are two particularly positive features of the new data:

1. On the one hand, only one of the anomalous bins in $P'_5$ ([4, 6]) sees its individual significance marginally decreased from $2.9\sigma$ to $2.7\sigma$, while the second one ([6, 8]) remains at $2.9\sigma$. However, the change in central value and uncertainty for $\langle P_5^\prime \rangle _{[4,6]}$ improves the agreement among the different observables, especially with $R_K$, for our most favoured NP scenarios, as illustrated in Fig. 2.
2. On the other hand, the new average value for $F_L$ in the bin [2.5, 4] is now more than $4\sigma$ below 1, while the previous value was at approximately $1\sigma$ from 1, which generated instability problems in some optimised observables in this bin due to a normalization. With the new data this problem is alleviated and we can use the optimised observables in all bins.

In summary, all results show now the following global picture:

- Besides an increase of significance of some scenarios (up to 0.8$\sigma$), there is no significant change, neither in the hierarchies among scenarios, nor in confidence intervals for the Wilson coefficients, with respect to the results presented in our earlier analysis presented in Ref. [1]. Our updated results therefore confirm the preexisting picture which calls for NP and they support the scenarios already favoured to explain the deviations.
- There is a reduction of the internal tensions between some of the most relevant observables of the fit, in particular, between the new averages of $R_K$ and $P'_5$. This leads to an increase in consistency between the different anomalies. This is illustrated in Fig. 2 (left) showing a better agreement between the predictions for $P_5^\prime$ in the most relevant NP scenarios and its updated measurement. Furthermore, in Fig. 2 (right), the best-fit points for the three favoured NP scenarios ${{\mathcal {C}}}^\mathrm{{NP}}_{9\mu }$ (Ref. [11]), $\{{{\mathcal {C}}}^\mathrm{{NP}}_{9\mu }, {{\mathcal {C}}}_{9'\mu }=-{{\mathcal {C}}}_{10'\mu }\}$ (Ref. [1]) and $\{{{\mathcal {C}}}^\mathrm{{V}}_{9\mu }=-\mathcal{C}^\mathrm{{V}}_{10\mu }, {{\mathcal {C}}}^\mathrm{{U}}_{9}\}$ (Ref. [2]) can explain two of the most relevant anomalies, $\langle P_5^\prime \rangle _{[4,6]}$ and $R_K$, in a perfect way. On the contrary, we see that the scenarios of NP in ${{\mathcal {C}}}_{10\mu }$ only or in ${{\mathcal {C}}}_{9\mu }^\mathrm{NP}=-{{\mathcal {C}}}_{10\mu }^\mathrm{NP}$ do not provide such a good agreement (this holds for any value of the NP contribution).
- The reduced uncertainties of the $B\rightarrow K^*\mu \mu$ data and its improved internal consistency sharpen statistical statements on the hypotheses considered. There is a significant increase of the statistical exclusion of the SM hypothesis as its p-value is reduced down to $1.4\%$ (i.e. 2.5$\sigma$). The $\hbox {Pull}_\mathrm{SM}$ of the 6D fit is now higher (5.8$\sigma$).
- Finally, we have updated the figures corresponding to specific simplified models in Fig. 5. In particular, our scenario 8 can still be interpreted in an EFT framework explaining $b\rightarrow c\ell \nu$ and $b\rightarrow s\ell \ell$ through correlated singlet and triplet dimension-6 operators combining quark and lepton bilinears. Both $b\rightarrow s\ell \ell$ and $b\rightarrow c\ell \nu$ show a very good agreement with this interpretation (see the right-hand side of Fig. 5) which indicates that scenario 8 is compatible with the tensions in $R_{D^{(*)}}$ if one assumes that the only significant contributions come from the operators ${{\mathcal {O}}}^{2333}$ and ${{\mathcal {O}}}^{2322}$ in the language of Ref. [12]. The pull of this scenario reaches 7.4$\,\sigma$ taking into account the deviations also observed in $R_{D^{(*)}}$.

The updated measurements of the $B\rightarrow K^*\mu \mu$ angular observables give also further possibilities to cross check the stability of our fits regarding internal inconsistencies within the data or underestimated hadronic effects by examining the $q^2$-dependence of our extraction (see Fig. 6). We perform fits testing 1D hypotheses selecting only the available LHCb data for $B\rightarrow K^*\mu \mu$ branching ratios and angular observables [3, 13, 14] in a given bin in $q^2$, together with data on $B_s\rightarrow \mu \mu$, $B\rightarrow X_s\mu \mu$ and $b \rightarrow s\gamma$ processes. We consider 1) the scenario with NP only in ${{\mathcal {C}}}_{9\mu }$, 2) the scenario with NP in $\mathcal{C}_{9\mu }^\mathrm{NP}=-{{\mathcal {C}}}_{10\mu }^\mathrm{NP}$, 3) the scenario 8, where we fix the LFUV part ${{\mathcal {C}}}_{9\mu }^\mathrm{V}=-\mathcal{C}_{10\mu }^\mathrm{V}$ to the b.f.p of the global fit and determine the value of ${{\mathcal {C}}}_{9}^\mathrm{U}$ through the fit. In all three cases, we observe an excellent agreement between the bin-by-bin determination and the outcome of the global fit, without significant $q^2$-dependence. For the scenario with NP only in $\mathcal{C}_{9\mu }$, a $q^2$-variation could have been the sign of underestimated hadronic effects from $c{\bar{c}}$-loop contributions [15]. For the two other scenarios, a $q^2$-dependence would have been the indication of an inconsistency in the experimental data or the theoretical approaches (in particular between the low- and large-recoil bins, where very different theoretical tools are used). It is very reassuring to see that there are no hints of such problems in our analyses.

In the future, we expect more data not only to reduce the uncertainties on the $B\rightarrow K^*\mu \mu$ observables, but also to increase further the consistency between $B\rightarrow K^*\mu \mu$ data and the rest of the data. On the basis of Figs. 2 and 6 , we see that several NP scenarios currently favoured by our global fit would push the central value of $\langle P'_5 \rangle _{[6,8]}$ slightly closer to the SM value than currently measured, whereas the determination of $P_5'$ in the other bins should yield the same central values as now.

In conclusion, we see that the recent update of $B \rightarrow K^*\mu \mu$ optimised observables by the LHCb collaboration leads to improved constraints on NP scenarios. The overall preferences for specific scenarios remain unchanged but we observe a higher consistency among the data analysed in the framework of the favoured scenarios. We expect thus the final update of both $B \rightarrow K^*\mu \mu$ optimised observables and $R_K$ including all the remaining recorded data to be an important step forward in the clarification of the *b*-flavour anomalies and the understanding of their origin.

## Data Availability

This manuscript has no associated data or the data will not be deposited. [Authors’ comment: The experimental data used in the analysis presented in this addendum has already been published by the different experimental collaborations cited in the manuscript. Any further data related to the specifics of our analysis can be reproduced by following the procedures detailed in the text and references therein, that we cite whenever it is appropriate.]

## Appendix: Correlations among fit parameters

In addition to the confidence regions provided for the various scenarios in this article, we display here the correlation matrices among the Wilson coefficients for the most interesting NP scenarios including the data available in March 2020.

### Correlation matrices of fits to LFUV NP

Following the same ordering for the correlation matrices as in Appendix A1 of Ref. [1], we find for the updated analysis:

$$\begin{aligned} \text {Corr}(\mathcal {C}_{9\mu }^\text {NP},\mathcal {C}_{10\mu }^\text {NP})= & {} \begin{pmatrix} 1.00&{}0.24\\ 0.24&{}1.00 \end{pmatrix}\\ \text {Corr}(\mathcal {C}_{9\mu }^\text {NP},\mathcal {C}_{9'\mu })= & {} \begin{pmatrix} 1.00&{}-0.35\\ -0.35&{}1.00 \end{pmatrix}\\ \text {Corr}(\mathcal {C}_{9\mu }^\text {NP},\mathcal {C}_{10'\mu })= & {} \begin{pmatrix} 1.00&{}0.32\\ 0.32&{}1.00 \end{pmatrix}\\ \text {Corr}(\mathcal {C}_{9\mu }^\text {NP},\mathcal {C}_{9e}^\text {NP})= & {} \begin{pmatrix} 1.00&{}0.47\\ 0.47&{}1.00 \end{pmatrix}\\ \text {Corr}(\mathcal {C}_{9\mu }^\text {NP}= & {} -\mathcal {C}_{9'\mu },\mathcal {C}_{10\mu }^\text {NP}=\mathcal {C}_{10'\mu })= \begin{pmatrix} 1.00&{}-0.18\\ -0.18&{}1.00 \end{pmatrix}\\ \text {Corr}(\mathcal {C}_{9\mu }^\text {NP},\mathcal {C}_{9'\mu }= & {} -\mathcal {C}_{10'\mu })= \begin{pmatrix} 1.00&{}-0.32\\ -0.32&{}1.00 \end{pmatrix} \end{aligned}$$

The last two matrices correspond to Hyp. 1 and Hyp. 5 in Table 3.

Regarding the 6D fit of Table 4,

$$\begin{aligned} \text {Corr}_\text {6D}= \begin{pmatrix} 1.00&{}-0.33&{}-0.06&{}0.04&{}0.04&{}0.01\\ -0.33&{}1.00&{}0.21&{}-0.04&{}0.02&{}0.22\\ -0.06&{}0.21&{}1.00&{}-0.12&{}0.53&{}0.52\\ 0.04&{}-0.04&{}-0.12&{}1.00&{}-0.14&{}-0.07\\ 0.04&{}0.02&{}0.53&{}-0.14&{}1.00&{}0.83\\ 0.01&{}0.22&{}0.52&{}-0.07&{}0.83&{}1.00 \end{pmatrix} \end{aligned}$$

where the columns in the matrix above are organized as in the analogous matrix in Appendix A1 of Ref. [1].

$\text {Corr}_\text {6D}$ shows a significant correlation in the pairs $\{\mathcal {C}_{10\mu }^\text {NP},\mathcal {C}_{9'\mu }\}$, $\{\mathcal {C}_{10\mu }^\text {NP},\mathcal {C}_{10'\mu }\}$ and $\{\mathcal {C}_{9'\mu },\mathcal {C}_{10'\mu }\}$, which is coherent with previous results in Appendix A1 of Ref. [1].

#### Correlation matrices of fits to LFUV-LFU NP

We also provide the correlations between fit parameters of scenarios 5–11 from Table 5, in that order:

$$\begin{aligned} \text {Corr}(\mathcal {C}_{9\mu }^\text {V},\mathcal {C}_{9}^\text {U}= & {} \mathcal {C}_{10}^\text {U},\mathcal {C}_{10\mu }^\text {V})= \begin{pmatrix} 1.00&{}-0.94&{}0.92\\ -0.94&{}1.00&{}-0.95\\ 0.92&{}-0.95&{}1.00 \end{pmatrix}\\ \text {Corr}(\mathcal {C}_{9\mu }^\text {V}= & {} -\mathcal {C}_{10\mu }^\text {V},\mathcal {C}_{9}^\text {U}=\mathcal {C}_{10}^\text {U})= \begin{pmatrix} 1.00&{}0.13\\ 0.13&{}1.00 \end{pmatrix}\\ \text {Corr}(\mathcal {C}_{9\mu }^\text {V},\mathcal {C}_{9}^\text {U})= & {} \begin{pmatrix} 1.00&{}-0.87\\ -0.87&{}1.00 \end{pmatrix}\\ \text {Corr}(\mathcal {C}_{9\mu }^\text {V}= & {} -\mathcal {C}_{10\mu }^\text {V},\mathcal {C}_{9}^\text {U})= \begin{pmatrix} 1.00&{}-0.46\\ -0.46&{}1.00 \end{pmatrix}\\ \text {Corr}(\mathcal {C}_{9\mu }^\text {V}= & {} -\mathcal {C}_{10\mu }^\text {V},\mathcal {C}_{10}^\text {U})= \begin{pmatrix} 1.00&{}0.71\\ 0.71&{}1.00 \end{pmatrix}\\ \text {Corr}(\mathcal {C}_{9\mu }^\text {V},\mathcal {C}_{10}^\text {U})= & {} \begin{pmatrix} 1.00&{}0.02\\ 0.02&{}1.00 \end{pmatrix}\\ \text {Corr}(\mathcal {C}_{9\mu }^\text {V},\mathcal {C}_{10'}^\text {U})= & {} \begin{pmatrix} 1.00&{}0.21\\ 0.21&{}1.00 \end{pmatrix} \end{aligned}$$

The situation is very similar to the previous analysis in Appendix A2 of Ref. [1], showing a high anti-correlation between $\mathcal {C}_{9\mu }^\text {V}$ and $\mathcal {C}_{9}^\text {U}$, and an even more reduced correlation between the coefficients of the scenario $\{\mathcal {C}_{9\mu }^\text {V},\mathcal {C}_{10}^\text {U}\}$.

## Footnotes

1 It can also be modified by contributions to scalar or pseudoscalar NP contributions, as well as by NP contributions with an imaginary part, but we do not consider such hypotheses among our NP scenarios.
